# Current practices and perceptions on diagnostic reference levels: a EuroSafe Imaging Survey Analysis

**DOI:** 10.1186/s13244-025-02028-z

**Published:** 2025-07-18

**Authors:** John Damilakis, Boris Brkljacic, Guy Frija, Timo De Bondt, Graciano Paulo, Virginia Tsapaki, Eliseo Vano, the European Society of Radiology (ESR)

**Affiliations:** 1 School of Medicine, University of Crete, Iraklion, Crete Greece; 2 School of Medicine, University of Zagreb, Šalata 3, 10000 Zagreb, Croatia; 3 Universite de Paris, 12 Rue de L’Ecole de Medecine, 75006 Paris, France; 4 VITAZ, Moerlandstraat 1, Sint-Niklaas, Belgium; 5 Medical Imaging and Radiotherapy Department, ESTESC-Coimbra Health School, Instituto Politecnico de Coimbra, 3046‑854 Coimbra, Portugal; 6 Medical Physics, Konstantopoulio General Hospital, Nea Ionia, Greece; 7 Complutense University, Madrid, Spain; 8 Am Gestade 1, Vienna, Austria

**Keywords:** Diagnostic reference levels, Optimisation, Radiation dosage, Surveys and questionnaires

## Abstract

**Abstract:**

Despite progress in implementing diagnostic reference levels (DRLs) across Europe, clinical practices remain variable. This prompts the EuroSafe Imaging campaign to conduct a survey assessing current practices, perceptions, and challenges related to DRLs. A total of 146 responses were collected from radiology departments in 38 countries, predominantly in the EU/EEA region. While 52.4% reported established local DRLs, significant gaps were identified, with 34.5% lacking local DRLs and 13.1% unaware of their existence. DRLs were primarily established for computed tomography (CT) (88.7%) and conventional radiography (77.5%), with lower implementation in interventional radiology (36.6%). Key challenges included time constraints, data collection difficulties, and limited standardization across institutions. Education gaps were notable, with less than half of the respondents reporting DRL-related training for radiology residents. Respondents emphasized the need for dose management systems, personalized DRLs based on clinical indications, and enhanced education and policy support. Addressing barriers through targeted training, policy enhancements, and technological innovations can improve DRL implementation. Future efforts should focus on promoting standardized clinical protocols, increasing awareness, and fostering European and international collaboration to ensure the consistent use and optimization of DRLs in clinical practice.

**Critical relevance statement:**

The article critically examines the variability and challenges in implementing diagnostic reference levels (DRLs) across European radiology departments, providing actionable recommendations on policy, education, and technological advancements to optimize radiation protection and improve clinical radiology practices.

**Patient summary:**

Diagnostic reference levels (DRLs) help healthcare providers ensure that radiation doses from medical imaging, like CT scans and X-rays, are not higher than necessary. This study looked at how DRLs are used across Europe. It found that while many hospitals have established and follow DRLs, others do not, which may affect patient safety. Challenges like time constraints and lack of training prevent better use of DRLs. Improving education for medical staff and updating protocols can help protect patients by reducing unnecessary radiation exposure while still ensuring accurate diagnoses.

**Key Points:**

- Variability persists in diagnostic reference level (DRL) practices across Europe.
- Over half of radiology departments have established local DRLs.
- Less than half of radiology residents receive structured DRL training.
- Improved DRL adoption can optimize radiation protection and patient safety.
- Collaboration, training, and standardized protocols are essential for better DRL practices.

**Graphical Abstract:**

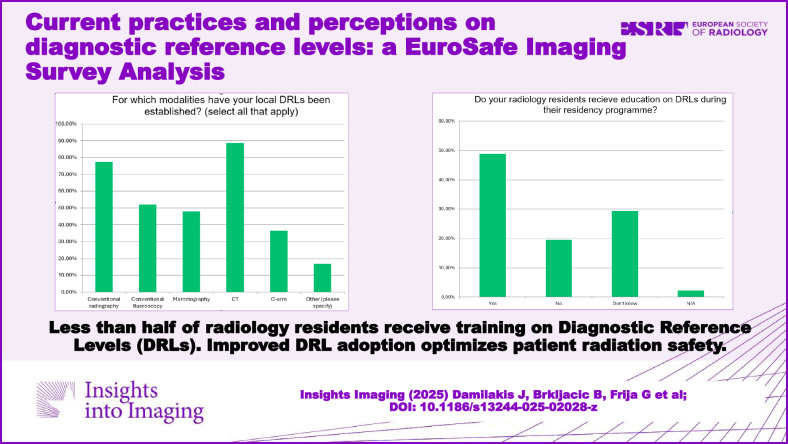

## Introduction

The European Commission’s Basic Safety Standards (BSS) Directive (Council Directive 2013/59/Euratom) mandates that all European Union (EU) Member States establish and implement Diagnostic Reference Levels (DRLs) as a cornerstone of optimizing medical imaging practices [1]. The primary goal of DRLs is not to define dose limits but to encourage the reduction of unnecessary exposure while maintaining adequate image quality for accurate diagnosis. This dual focus ensures that patient safety is prioritized without compromising the diagnostic efficacy of medical imaging procedures [2, 3].

As a benchmarking tool, DRLs allow healthcare facilities to compare their reference levels against local, national or regional standards and identify significant deviations. These deviations may indicate excessive radiation exposure or inefficiencies in imaging protocols, necessitating targeted corrective actions to improve practices [4]. Research underscores the effectiveness of DRLs in mitigating dose variations and achieving meaningful dose reductions without impairing diagnostic accuracy [5]. By fostering consistency and safety in radiological practices, DRLs play a pivotal role in advancing radiation protection and optimizing patient care in medical imaging.

The European Union has made significant progress in transposing the BSS Directive into national legislation. Member States have established national DRLs for a wide range of examinations, including computed tomography (CT), plain radiography, mammography, fluoroscopy, and nuclear medicine procedures [6, 7]. To maintain their effectiveness as tools for radiation protection and optimization, these national DRLs require regular review and updates to reflect technological advancements, variations in clinical practices, and changes in patient demographics. This iterative approach ensures that DRLs remain relevant and continue to drive optimization efforts effectively [8].

To support the implementation of DRLs, key initiatives such as the “Pediatric DRL” project and the EUCLID (European Study on Clinical Diagnostic Reference Levels for X-ray Medical Imaging) project, funded by the European Commission, have been launched [6, 9]. The European guidelines on DRLs for pediatric imaging (PiDRL) provide specific recommendations for optimizing pediatric imaging protocols, addressing the unique sensitivity of children to ionizing radiation. The EUCLID project focused on establishing clinical DRLs for adult CT examinations and fluoroscopically guided procedures. These specific procedures were prioritized due to their high frequency and significant contribution to the collective radiation dose received by the population, highlighting the critical need for their optimization.

The process of harmonization of DRL frameworks across Europe has significantly advanced international benchmarking, enabling the comparison of dose levels between countries and fostering the adoption of best practices in dose management. However, despite the widespread implementation of DRLs, a comprehensive assessment of the practices and perceptions surrounding their establishment and use has been lacking. To address this gap, the EuroSafe Imaging campaign, dedicated to promoting radiation protection and safety in medical imaging, established a working group on clinical DRLs. This group conducted a survey to examine current practices and perceptions related to DRLs, providing valuable insights into their application in clinical settings and identifying opportunities for improvement. The findings of this survey aim to contribute to a deeper understanding of DRL implementation and inform future optimization efforts.

## Material and methods

### Survey development and distribution

A survey was developed by the EuroSafe Imaging working group on clinical DRLs to gather information on the establishment and use of DRLs in clinical settings. Respondents were encouraged to complete the survey in consultation with the head of their Radiology Department, with the option to delegate this task to a medical physicist or radiographer if deemed appropriate. The survey was administered via the online platform SurveyMonkey. Any personal data provided, including names, email addresses, and institutional affiliations, was used solely for data verification purposes within ESR EuroSafe Imaging. All survey data were analyzed and reported in an aggregate format, with strict attention paid to ensuring anonymity. Care was taken to ensure that no individual or specific organization could be identified in the survey results. Ethical approval was not required as the study involved anonymized data and did not include any direct patient involvement.

### Survey questions

The questionnaire consisted of 34 multiple-choice and open-field questions presented in English. The questions and their corresponding response options are available in the supplementary material. The questionnaire was divided into four sections: the first collected background information about the respondents and their institutions; the second focused on the use and implementation strategies of local DRLs; the third explored healthcare professionals’ perceptions of DRLs; and the fourth solicited recommendations for policy changes, educational requirements, and future developments in DRL practices.

### Statistics

To explore the relationship between educational factors and the implementation of local DRLs, we performed chi-square (*χ*²) tests of independence. Associations were evaluated between DRL implementation status and provision of DRL-related training for radiology residents. All tests were conducted using two-sided significance with a *p*-value threshold of < 0.05. Statistical analysis was performed using MedCalc (MedCalc Software Ltd, version 23.2.1).

## Results

### Survey response demographics

A total of 146 responses were collected from radiology departments in 38 countries. Of these, 105 (71.9%) originated from European Union /European Economic Area (EU/EEA) countries, while the remaining 41 (28.1%) were from non-EU/EEA countries. A considerable proportion of responses were received from Italy (18, 12.3%), Germany (11, 7.5%), and Spain (11, 7.5%).

### Gender distribution and professional background

The respondents of the survey were 60.9% male, 37.9% female, while the rest were identified as others or preferred not to disclose. This gender imbalance may reflect the gender distribution in the professions surveyed. Most respondents were radiologists (74.0%), followed by fewer medical physicists and radiographers. As most insights come from radiologists, the results may be skewed toward their experiences and practices. Additionally, 38.4% of respondents had over 20 years of experience, indicating that many respondents represent seasoned professionals. This is valuable as it reflects long-term practices and potentially established views on DRLs.

### Institutional characteristics

Most respondents (69.9%) worked in public institutions, with 37.0% employed in large institutions (over 800 beds). This suggests that DRL practices are more prevalent in larger institutions with high patient throughput and imaging volume.

### Knowledge and use of DRLs

Respondents reported a relatively high level of knowledge about DRLs, with 35.0% indicating high knowledge, 34.9% medium, 22.6% low, and 7.5% unaware of DRLs. Over half (52.4%) had established local DRLs, but the remaining 34.5% (no) and 13.1% (don’t know) highlighted gaps in DRL implementation. DRLs were routinely used by 74.6%, with occasional or rare use reported by smaller percentages.

### Modalities and applications of DRLs

**Fig. 1:**
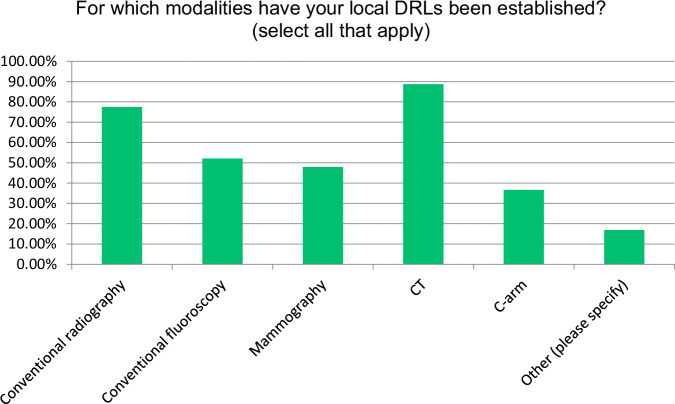
Distribution of imaging modalities with established local DRLs

**Fig. 2:**
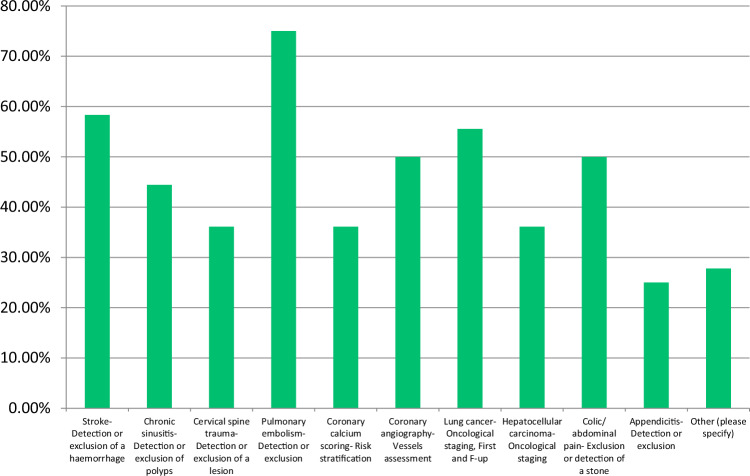
Most common clinical indications for CT with established local DRLs

**Fig. 3:**
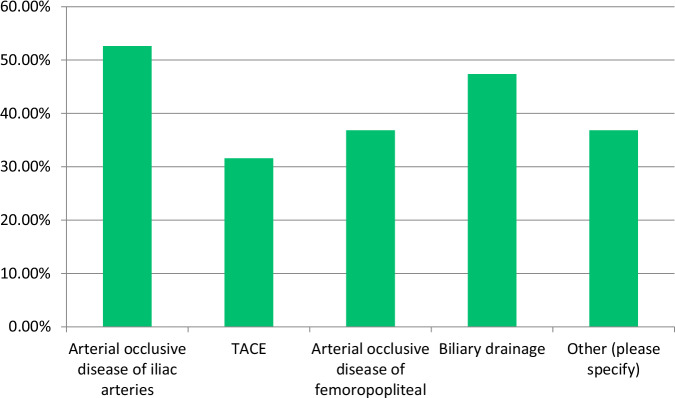
Clinical indications for interventional radiology with established DRLs. TACE, transarterial chemoembolization

The most common modalities with established local DRLs were CT (88.7%) and conventional radiography (77.5%), followed by fluoroscopy and mammography (Fig. 1). DRL usage in interventional radiology was less frequent (36.6%). Pulmonary embolism (75.0%) and stroke (58.3%) were the most common CT clinical indications, reflecting the diagnostic importance of CT in these conditions (Fig. 2). In interventional radiology, arterial occlusive disease of the iliac arteries (52.6%) was the most cited clinical indication (Fig. 3). 50.7% of respondents base their DRLs on both anatomy and clinical indications, indicating a comprehensive approach. However, the split between purely anatomy-based (35.2%) and clinical indication-based (14.1%) approaches may reflect variability in DRL practices.

### Tools and benchmarking practices

A significant proportion (80.4%) use dose management systems (DMS) to implement local DRLs. This high percentage of respondents utilizing DMS for local DRLs suggests that this sample may not be fully representative of all institutions, as many hospitals in Europe may not be using DMS for dose monitoring. However, this finding indicates that where local DRLs are used, they are often supported by dedicated tools, which is a positive sign for effective dose monitoring. Only 41.1% compare local DRLs with those from other institutions, suggesting an opportunity for improvement in terms of collaboration and benchmarking. However, 85.7% compare their local DRLs with national ones, reflecting strong alignment with broader regulatory or professional guidelines. Monthly reference to local DRLs (37.5%) was most common, with fewer institutions using DRLs daily (19.6%). This suggests that local DRLs are used regularly but may not be fully integrated into daily practice across all institutions. Half of the respondents (50%) update their local DRLs yearly, which is good practice. The fact that some update less frequently (35.7%) or only after equipment renewal (14.3%) suggests that there may be barriers to more frequent updating, possibly related to resource limitations.

A high percentage (73.2%) of respondents use local DRLs to track outliers, which suggests that local DRLs are being utilized not only as a regulatory tool but also as a quality improvement measure. Slightly more than half of the respondents (53.6%) have had their local DRLs utilized by their competent authority for national DRLs, showing that local efforts are feeding into national practices, but with some respondents still unsure or uninvolved in this process.

### Motivation and confidence in using DRLs

The main motivation for using DRLs is optimization (67.9%), which is a key goal of DRLs. Regulatory compliance (25%) is also important, but there seems to be less interest in DRLs for research purposes (1.8%). Most respondents find DRLs either very useful (34.7%) or moderately useful (43.9%) in clinical practice, confirming that they are seen as a valuable tool for optimizing radiation doses. Confidence levels in interpreting and applying local DRLs in respondents’ work vary, with 33.7% being very confident and 39.8% moderately confident. However, 26.5% not feeling confident indicates a need for more education and training on DRLs.

### Education and training

Less than half (48.9%) reported that their radiology residents receive education on DRLs, highlighting an area for potential improvement in residency training programs. Continuing Professional Development (CPD) activities related to DRLs are lacking in many departments (53.3%), suggesting that ongoing education about DRLs could be expanded to improve knowledge and implementation.

Chi-square test revealed statistically significant associations between DRL implementation and provision of training. Institutions offering DRL education to radiology residents were significantly more likely to have established local DRLs (*χ*² = 7.48, *p* = 0.006). This finding highlights the critical role of education in supporting medical radiation protection practices.

### Challenges in implementing DRLs

The main barriers or challenges related to using local DRLs include time constraints due to busy departmental workloads, data collection and data cleaning challenges, misunderstandings about the statistical basis of DRLs, standardization issues among protocols across different hospitals, and limited awareness of DRL significance. These barriers highlight a mix of practical, technological, and educational challenges in the implementation of local DRLs in clinical practice.

### Suggestions for enhancing DRL practices

Respondents proposed several measures to improve DRL use: involving more staff in data collection and calculation of DRLs, developing and implementing comprehensive training programs for staff, increasing awareness among healthcare professionals regarding DRLs, aligning CT protocols with specific clinical indications, standardizing protocols across equipment and facilities, implementing DMS, conducting regular meetings to discuss DRL implementation and outcomes, and emphasizing quality control in imaging practices. These proposals reflect a combination of technical, educational, and administrative strategies to enhance the practical application of DRLs.

### Policy recommendations

Respondents highlighted policy changes needed to support DRL implementation: introducing mandatory DRL-related courses, increasing resources for Medical Physics Experts, incentivizing compliance and penalizing non-compliance, encouraging competent authorities to allocate more resources to DRL-related efforts, establishing workgroups to focus on DRL policy and implementation, and extending DRL applications to areas such as imaging in radiotherapy. These suggestions emphasize the need for stricter regulations, enhanced education, and better resource allocation to improve DRL practices.

### Future development in DRL practices

Respondents advocated for innovations such as DMS for all hospitals, installation of DRL-related tools in CT scanners, improved alert systems for CT exams when DRLs are exceeded, and development of personalized DRLs accounting for individual patient characteristics. Respondents also emphasized the importance of establishing DRLs tailored to specific clinical indications, particularly in CT, interventional radiology and cardiology, and the establishment of national DRLs focused on pediatric examinations. Some answers were focused on transparency and communication matters, such as including DRL information in dosimetric reports provided to patients and promoting better understanding of the pros and cons of DRLs among professionals. Some respondents focused on improved data management, e.g., more frequent reviews and updates of DRL data. These responses reflect the need for innovation in technology, personalization of DRLs, and improvements in policies and communication to advance DRL practices.

### Continuing professional development and training

Mandatory courses on ionizing radiation safety and structured teachings, including case discussions and protocol optimizations, were recommended as part of regular CPD activities. However, existing training materials were noted to lack depth or engagement, highlighting the need for enhanced educational resources.

## Discussion

The findings of this survey provide valuable insights into the current practices and perceptions surrounding DRLs across radiology departments. Despite significant advancements in DRL implementation, the results reveal critical areas for improvement and highlight opportunities for innovation, policy enhancement, and educational initiatives.

### DRL implementation and usage

The survey indicates that over half (52.4%) of the respondents have established local DRLs, and DRLs are routinely used by 74.6%. However, the variability in DRL implementation highlights significant gaps, with 34.5% reporting no local DRLs and 13.1% unsure of their existence. This suggests that while DRLs are widely recognized as essential tools for optimization of radiation protection, their adoption is not yet universal. Previous studies have similarly emphasized that DRLs, while effective in reducing unnecessary radiation exposure, require consistent updates and broader implementation to address variations in clinical practices [8, 9].

A noteworthy finding is that 53.6% of respondents reported their local DRLs being utilized by competent authorities to inform national DRLs, demonstrating that local efforts are contributing to broader national practices. However, this also highlights that a considerable proportion of institutions remain uninvolved or unaware of how their data feeds into national frameworks. Such disengagement suggests missed opportunities for collaboration and underscores the need for more structured communication channels between local facilities and national regulatory bodies.

### Modalities and clinical indications

The predominant use of DRLs in modalities such as CT (88.7%) and conventional radiography (77.5%) aligns with global trends [10–12]. The lower implementation rates in interventional radiology (36.6%) and other specialized imaging procedures suggest a need to expand DRL applications to procedures with high frequency and high patient radiation exposure [13].

### Challenges in DRL implementation

Barriers identified in this survey, including time constraints, data collection challenges, and standardization issues, are consistent with those reported in other studies [8]. The lack of awareness and understanding of the statistical basis of DRLs among professionals further complicates their implementation. Addressing these challenges will require targeted education and resource allocation to streamline DRL integration into clinical workflows.

### Education and training gaps

The survey highlights substantial gaps in education and training, with only 48.9% of respondents reporting that radiology residents receive DRL-related education. CPD activities were also lacking in over half of the departments (53.3%). As DRLs are a cornerstone of radiation protection, it is imperative to integrate comprehensive DRL education into residency training and CPD programs [14]. This is emphasized also in the EU-REST project dealing with education issues about radiation protection and continuous professional development of professionals dealing with ionizing radiation in the EU (https://www.eurosafeimaging.org/eu-rest). Enhanced training initiatives can improve confidence in interpreting and applying DRLs, as reflected in the 26.5% of respondents who reported a lack of confidence.

### Future directions for DRL development

Respondents emphasized the need for personalized DRLs that account for individual patient characteristics and clinical indications. Additionally, the integration of advanced technologies, such as DMS and improved data-sharing mechanisms, was identified as a priority [15, 16]. DMS can facilitate more frequent updates of DRLs and enable their seamless integration into clinical practice. These findings align with recent trends advocating for the refinement of DRLs to enhance their clinical relevance [8, 17].

### Policy recommendations

The survey findings underscore the need for robust policies to support DRL implementation. A critical suggestion involves introducing mandatory education and training programs on DRLs, both as part of residency curricula and ongoing CPD activities for all healthcare professionals involved in medical imaging. This would help address the gaps in awareness and confidence reported by respondents. Another key recommendation is the allocation of additional resources, such as providing funding for DMS, which would streamline the collection, analysis, and regular updating of DRL data. Regulatory measures, including incentives for compliance and penalties for non-compliance, were proposed to encourage adherence to DRL guidelines and best practices. Respondents also advocated for the establishment of dedicated workgroups focused on DRL policy development and implementation, which could enhance collaboration and standardization across institutions and regions. Expanding the application of DRLs to include emerging areas like radiotherapy imaging and pediatric-specific procedures was also emphasized, reflecting the need to tailor radiation protection strategies to diverse clinical contexts and vulnerable patient populations. Furthermore, transparent communication practices, such as including DRL data in reports provided to patients, were suggested to improve understanding and trust among patients and professionals. These policy recommendations collectively highlight the need for a coordinated approach that integrates education, technological innovation, resource allocation, and regulatory enforcement to optimize the use of DRLs in clinical practice and ensure patient safety.

### Limitations

This study has its limitations. Firstly, the survey’s primary focus on European countries restricts the generalizability of findings to other global regions with differing healthcare systems and radiation protection frameworks. While the findings reflect European practices, they may not adequately represent regions with limited resources or varying regulatory requirements. Secondly, the reliance on self-reported data introduces potential biases, such as over- or under-reporting of DRL implementation practices and challenges. The survey predominantly captured the perspectives of radiologists, which could skew the results towards the experiences and practices of this professional group, potentially underrepresenting the contributions and challenges faced by medical physicists and radiographers. Another limitation lies in the variability of institutional sizes and resources. A large proportion of responses came from public institutions and larger facilities. This may not reflect the challenges faced by smaller or private institutions with limited access to DMS or training resources.

## Conclusion

This study highlights both the achievements and the challenges in the implementation of DRLs. While DRLs are widely recognized as essential tools for optimization, their integration into routine clinical practice remains inconsistent. Addressing the identified gaps through education, policy enhancement, and technological innovation can significantly improve DRL adoption and effectiveness. These efforts will ultimately contribute to enhanced patient safety and optimized radiation practices in medical imaging.

One key finding of this survey is the critical role of DMS in supporting the effective use of DRLs. Proper utilization of DMS can streamline data collection, enable real-time monitoring, and facilitate compliance with DRLs.

Furthermore, the survey underscores the need to reinforce training on DRLs, particularly for radiology residents. Comprehensive training programs tailored to the specific needs of residents and other healthcare professionals are essential to ensure confident interpretation and application of DRLs in clinical practice. Targeted education initiatives can also help address knowledge gaps and foster a culture of radiation safety.

## Supplementary information

ELECTRONIC SUPPLEMENTARY MATERIAL

## Data Availability

Summary data from the survey is presented within the manuscript and accompanying figures.

## Abbreviations

BSS: Basic safety standards
CPD: Continuing professional development
CT: Computed tomography
DMS: Dose management systems
DRLs: Diagnostic reference levels
EUCLID: European Study on Clinical Diagnostic Reference Levels for X-ray Medical Imaging
