# Supplementary material for: Current practices and perceptions on diagnostic reference levels: a EuroSafe Imaging Survey Analysis
Source: Insights Imaging. 2025 Jul 18;16:156. doi: 10.1186/s13244-025-02028-z (PMC12274150; doi:10.1186/s13244-025-02028-z)
Supplement: Supplementary file 1 — ELECTRONIC SUPPLEMENTARY MATERIAL [file 13244_2025_2028_MOESM1_ESM.pdf]

# Current Practices and Perceptions on Diagnostic Reference Levels: A EuroSafe Imaging Survey Analysis

## ELECTRONIC SUPPLEMENTARY MATERIAL

Insights Imaging (2025) Damilakis J, Brkljacic B, Frija G, et al.

# Survey results

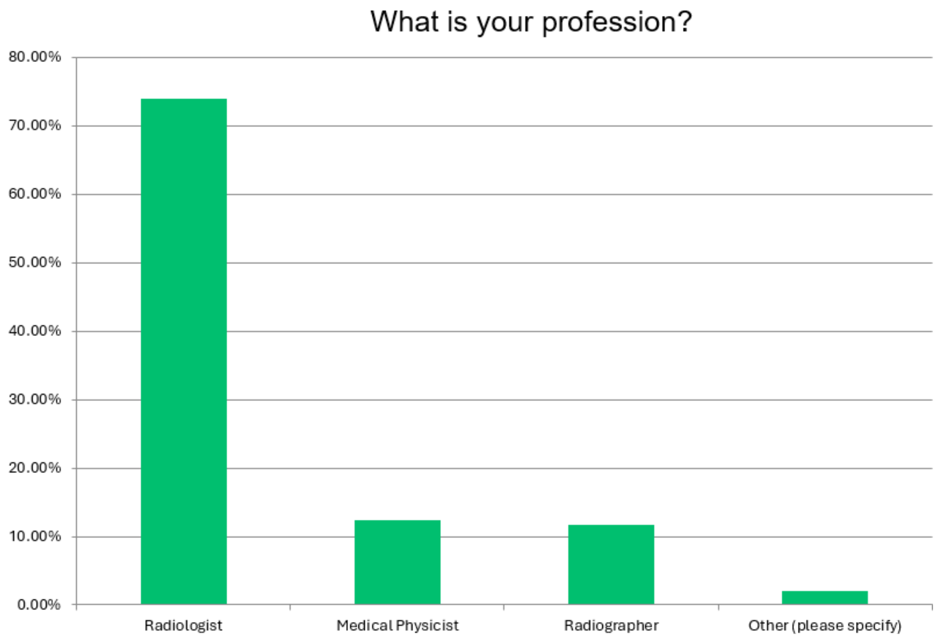


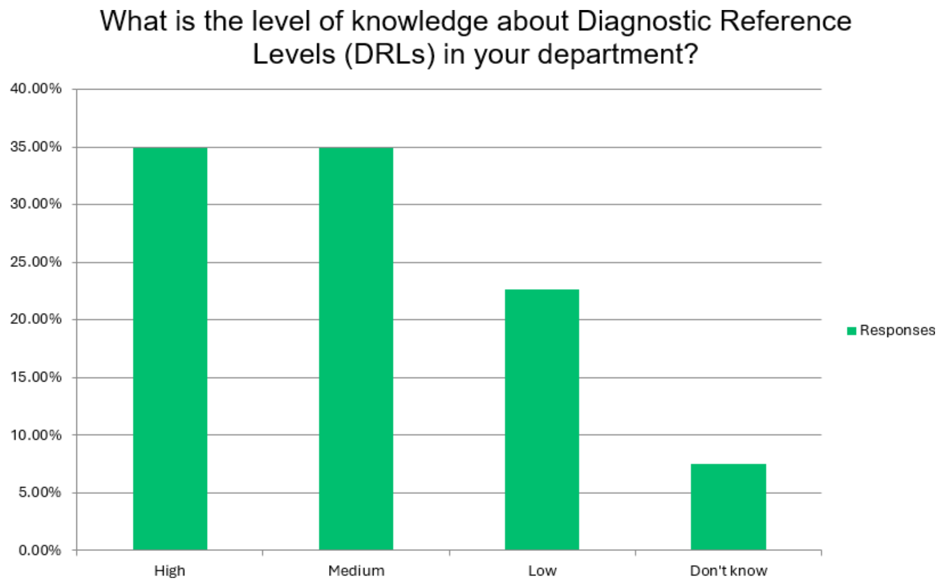

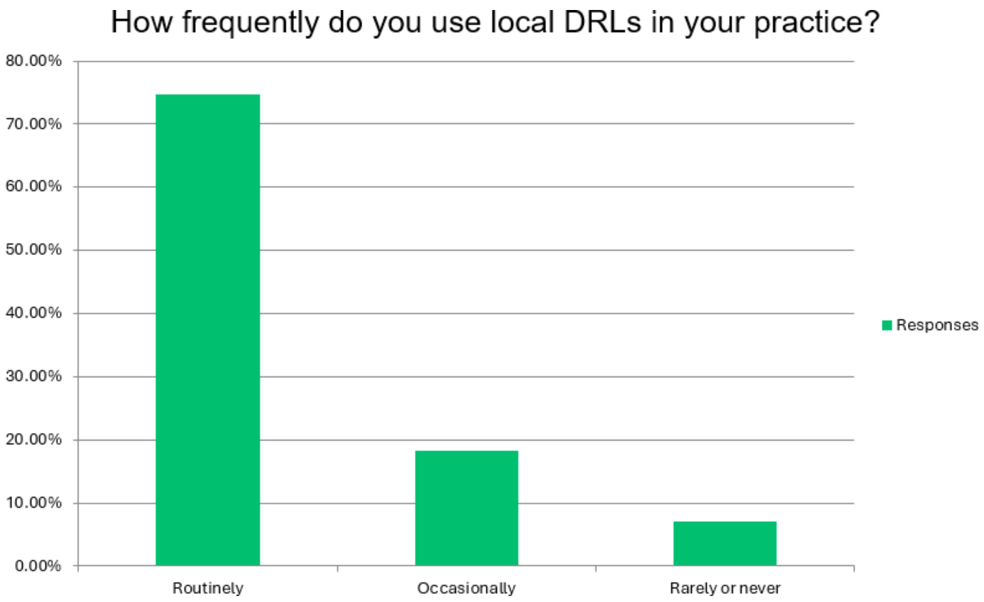
How frequently do you use local DRLs in your practice?
80.00%
70.00%
60.00%
50.00%
40.00%
30.00%
20.00%
10.00%
0.00%
Routinely
Occasionally
Rarely or never
Responses

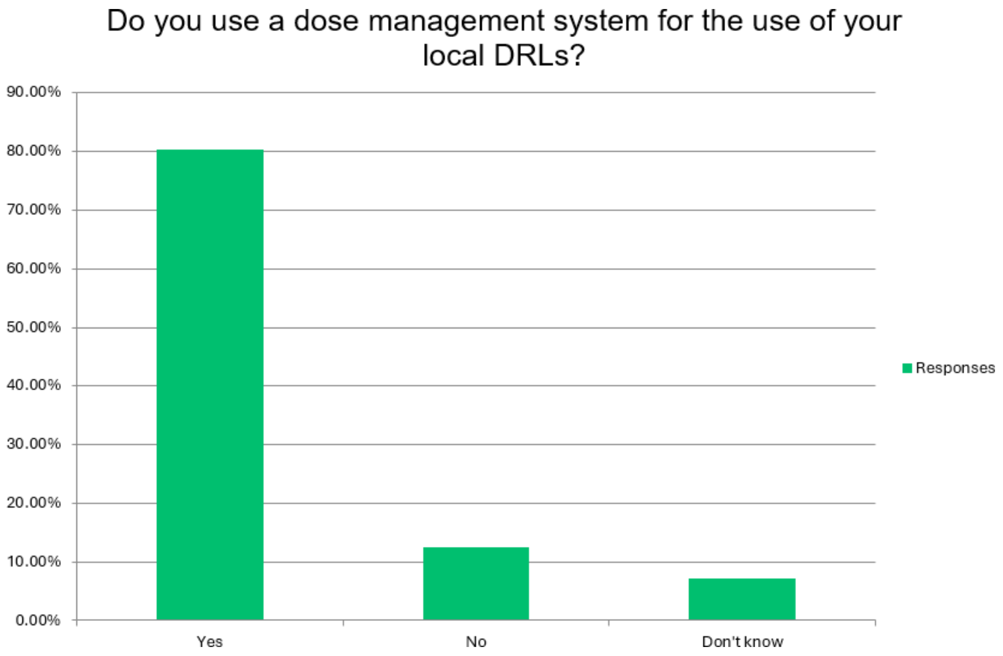
Do you use a dose management system for the use of your local DRLs?
90.00%
80.00%
70.00%
60.00%
50.00%
40.00%
30.00%
20.00%
10.00%
0.00%
Yes
No
Don't know
Responses

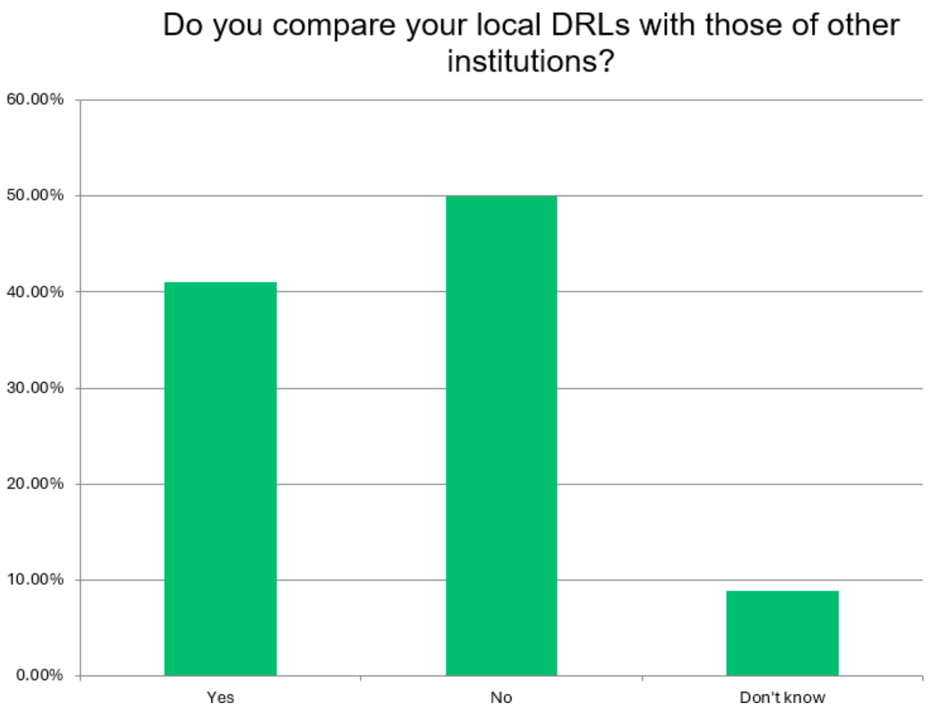
Do you compare your local DRLs with those of other institutions?
60.00%
50.00%
40.00%
30.00%
20.00%
10.00%
0.00%
Yes
No
Don't know

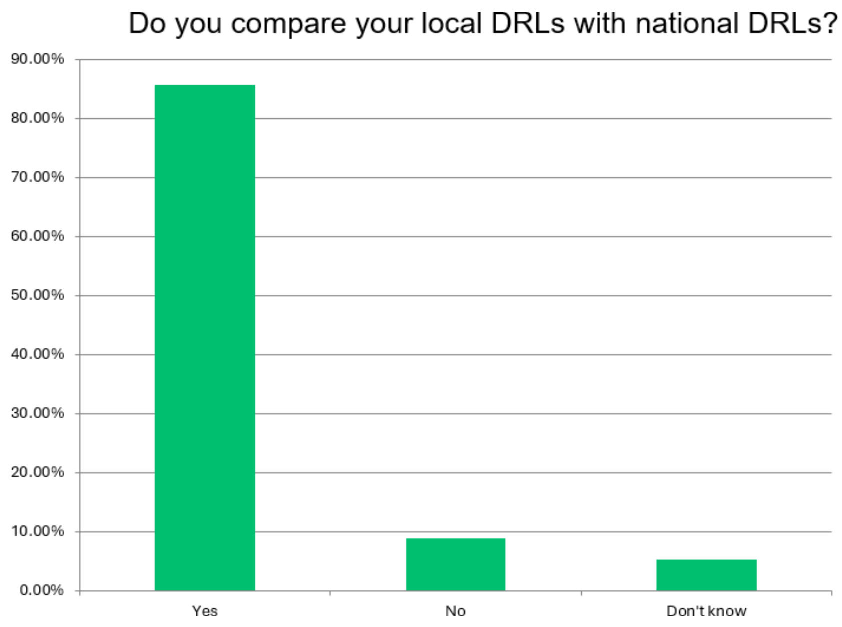
Do you compare your local DRLs with national DRLs?
90.00%
80.00%
70.00%
60.00%
50.00%
40.00%
30.00%
20.00%
10.00%
0.00%
Yes
No
Don't know

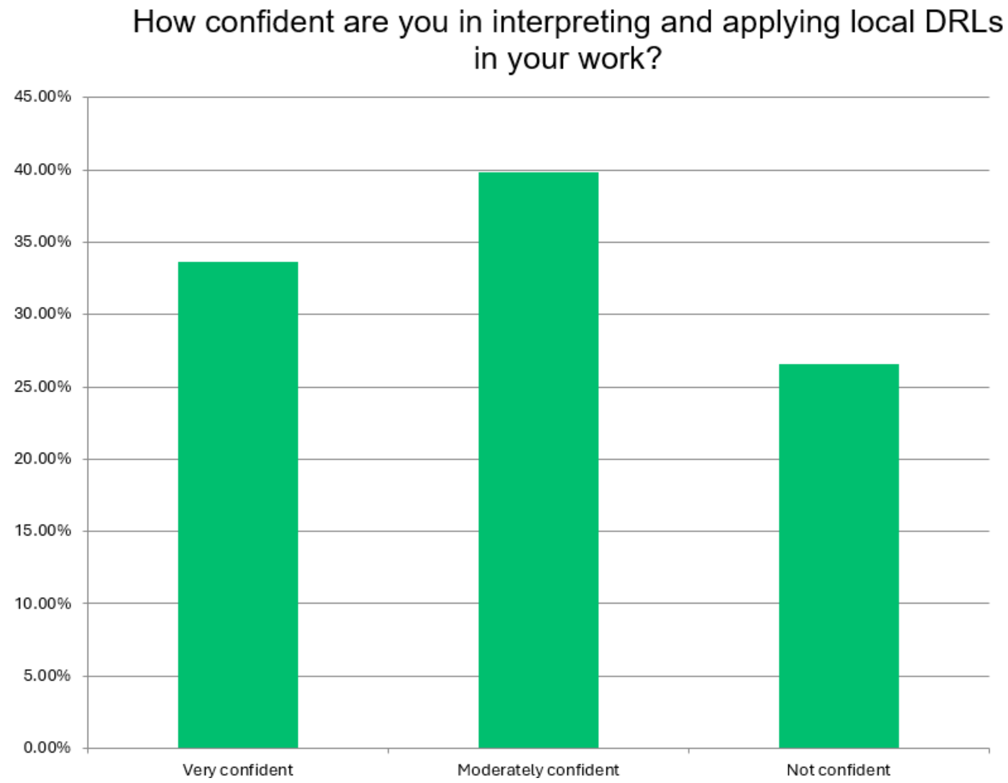
How confident are you in interpreting and applying local DRLs in your work?
45.00%
40.00%
35.00%
30.00%
25.00%
20.00%
15.00%
10.00%
5.00%
0.00%
Very confident
Moderately confident
Not confident

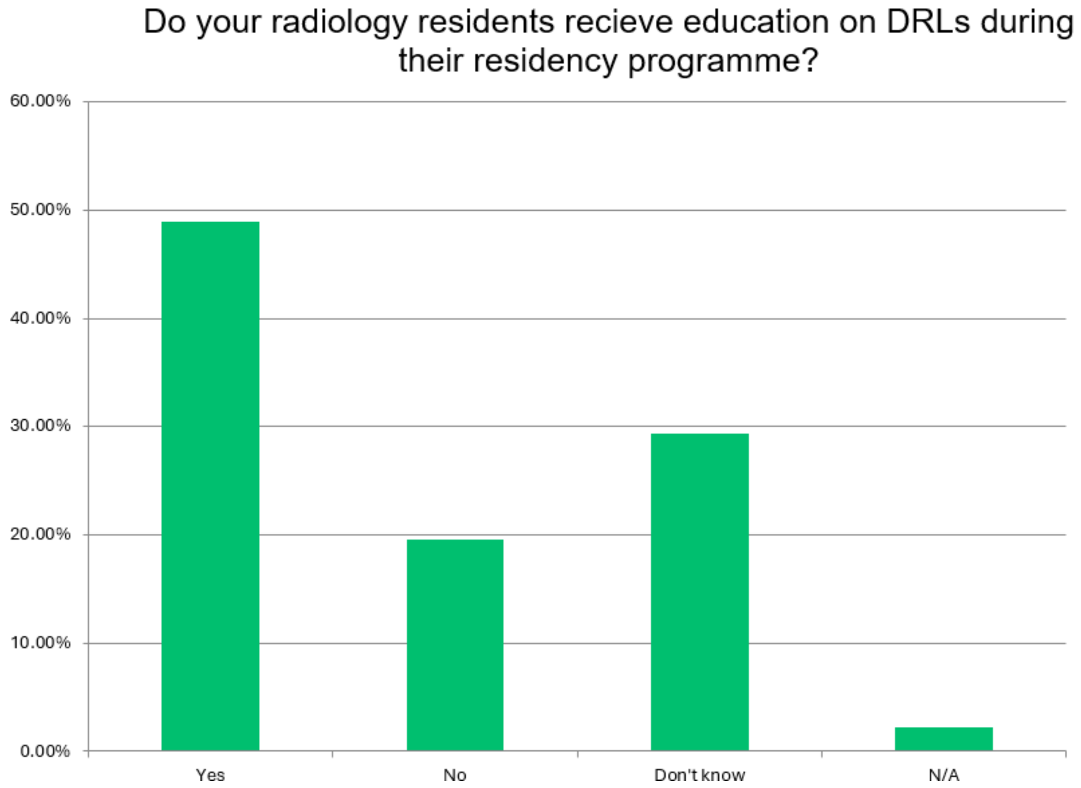
Do your radiology residents recieve education on DRLs during their residency programme?
60.00%
50.00%
40.00%
30.00%
20.00%
10.00%
0.00%
Yes
No
Don't know
N/A

## Background Information

This survey is designed to collect information about the use of local Diagnostic Reference Levels (DRLs). The questionnaire is structured into four sections: the first gathers background information on respondents and their institutions; the second assesses the usage and implementation strategies of local DRLs; the third section explores the perceptions of healthcare professionals regarding DRLs; and the fourth solicits recommendations for policy changes, educational needs, and future developments in DRL usage. Please complete this survey in consultation with your head of the Radiology Department. If appropriate, you can delegate this task to a medical physicist or radiographer. Your participation will contribute valuable data towards the effective use of DRLs in clinical practice, supporting both quality improvement and patient care optimisation.

**Data Protection, data processing and consent to participate**
The ESR EuroSafe Imaging working group on Clinical DRLs is responsible for fielding the survey and analysing the survey data. This survey is run via the online survey tool Survey Monkey. The personal data provided (your name, email adress, and name of institution) is used only for data verification purposes within ESR EuroSafe Imaging. All survey data will be reported in aggregate format. Special attention will be paid to ensuring that individuals or specific organisations cannot be traced back in the reported survey results. With the submission of your data/ information you agree to these terms.

* 1. Basic information

First name

Last name

Name of institution/hospital

Name of department

Age

2. What is your gender?

* 3. Email address

* 4. What is your profession?

- Radiologist
- Medical Physicist
- Radiographer
- Other (please specify)

* 5. How many years have you been working in your current role?

- <1
- 1-5
- 6-10
- 11-15
- 16-20
- >20

* 6. Do you work in a public or private medical setting?

- Public
- Private
- Both

* 7. What is the approximate number of beds in your institution?

- >800
- 500-800
- 200-500
- <200
- Not applicable

* 8. In which country are you currently practicing?

* 9. What is the level of knowledge about Diagnostic Reference Levels (DRLs) in your department?

- High
- Medium
- Low
- Don't know

## Usage of DRLs

* 10. Have you established local DRLs in radiology?

- ◯ Yes
- ◯ No
- ◯ Don't know

## Usage of DRLs

* 11. How frequently do you use local DRLs in your practice?

- ◯ Routinely
- ◯ Occasionally
- ◯ Rarely or never

* 12. For which modalities have your local DRLs been established? (select all that apply)

- ☐ Conventional radiography
- ☐ Conventional fluoroscopy
- ☐ Mammography
- ☐ CT
- ☐ C-arm
- ☐ Other (please specify)

* 13. Are your local DRLs based on anatomy or clinical indications?

- ◯ Anatomy
- ◯ Clinical indications
- ◯ Both

## For what clinical indications have you established local DRLs?

14. Computed Tomography- CT

- [ ] Stroke- Detection or exclusion of a haemorrhage
- [ ] Chronic sinusitis- Detection or exclusion of polyps
- [ ] Cervical spine trauma- Detection or exclusion of a lesion
- [ ] Pulmonary embolism- Detection or exclusion
- [ ] Coronary calcium scoring- Risk stratification
- [ ] Coronary angiography- Vessels assessment
- [ ] Lung cancer- Oncological staging, First and F-up
- [ ] Hepatocellular carcinoma- Oncological staging
- [ ] Colic/ abdominal pain- Exclusion or detection of a stone
- [ ] Appendicitis- Detection or exclusion
- [ ] Other (please specify)

15. Interventional Radiology- IR

- [ ] Arterial occlusive disease of iliac arteries
- [ ] TACE
- [ ] Arterial occlusive disease of femoropopliteal
- [ ] Biliary drainage
- [ ] Other (please specify)

16. All other modalities

## Usage of DRLs

* 17. Do you use a dose management system for the use of your local DRLs?

- Yes
- No
- Don't know

* 18. Do you compare your local DRLs with those of other institutions?

- Yes
- No
- Don't know

* 19. Do you compare your local DRLs with national DRLs?

- Yes
- No
- Don't know

* 20. Do you use your local DRLs as a quality benchmark to track outliers?

- Yes
- No
- Don't know

* 21. Do you use your local DRLs for purposes other than those described above?

- No
- Yes (please specify)

* 22. Have your local DRLs been utilized by your competent authority for the establishment of national DRLs?

- Yes
- No
- Don't know

* 23. How often do you refer to local DRLs in your work?

- ◯ Daily
- ◯ Weekly
- ◯ Monthly
- ◯ Yearly
- ◯ Never

* 24. How frequently do you update your local DRLs?

- ◯ Yearly
- ◯ Every two years
- ◯ Every three years
- ◯ Every four years
- ◯ Every five years
- ◯ Less often than every five years
- ◯ After equipment renewal or similar milestone

* 25. What was your **main** motivation for establishing and using local DRLs?

- ◯ Dose optimization
- ◯ Regulatory compliance
- ◯ Research
- ◯ Don't know
- ◯ Other (please specify)

## Perceptions

* 26. How useful do you find local DRLs in clinical practice?

- ◯ Very useful
- ◯ Moderately useful
- ◯ Not useful
- ◯ Don't know

* 27. How confident are you in interpreting and applying local DRLs in your work?

- ◯ Very confident
- ◯ Moderately confident
- ◯ Not confident

28. What are the three main barriers or challenges you face in using local DRLs?

| | |
|---|---|
| Barrier 1 | |
| Barrier 2 | |
| Barrier 3 | |

29. How could the use of DRLs in clinical practice be improved? Please provide up to three proposals

| | |
|---|---|
| Proposal 1 | |
| Proposal 2 | |
| Proposal 3 | |

## Recommendations and CPD activities

30. What policy changes would support the effective use of DRLs in clinical practice?

31. What future developments in DRLs would you like to see?

* 32. Do your radiology residents recieve education on DRLs during their residency programme?

- Yes
- No
- Don't know
- N/A

* 33. Are there other Continuing Professional Development (CPD) activities related to DRLs within your department?

- Yes
- No

34. Please describe the Continuing Professional Development (CPD) activities related to DRLs within your department:
